# Nitric Oxide as a Biomarker of Intracellular *Salmonella* Viability and Identification of the Bacteriostatic Activity of Protein Kinase A Inhibitor H-89

**DOI:** 10.1371/journal.pone.0058873

**Published:** 2013-03-15

**Authors:** Haiqi He, Kenneth J. Genovese, Christina L. Swaggerty, David J. Nisbet, Michael H. Kogut

**Affiliations:** Southern Plains Agricultural Research Center, USDA-ARS, College Station, Texas, United States of America; Bauer Research Foundation, United States of America

## Abstract

*Salmonella enterica* serovar Enteritidis is one of the most prevalent *Salmonella* serovars in poultry and is often associated with human salmonellosis. *S.* Enteritidis is known to suppress nitric oxide (NO) production in infected chicken macrophage HD11 cells, while dead *S.* Enteritidis stimulates a high level of NO production, suggesting a bacterial inhibitory effect on NO production. Based on these observations, the present study was conducted to evaluate whether NO production in *S.* Enteritidis-infected HD11 cells can be used as a biomarker to identify molecules that kill intracellular *Salmonella*. Since *Salmonella* are known to manipulate the host cell kinase network to facilitate intracellular survival, we screened a group of pharmaceutical inhibitors of various kinases to test our hypothesis. A protein kinase A inhibitor, H-89, was found to reverse the suppression of NO production in *S.* Enteritidis-infected HD11 cells. Production of NO in *S.* Enteritidis-infected HD11 cells increased significantly following treatment with H-89 at or above 20 µM. Inversely, the number of viable intracellular *Salmonella* decreased significantly in cells treated with H-89 at or above 30 µM. Furthermore, the growth rate of *S.* Enteritidis in culture was significantly inhibited by H-89 at concentrations from 20 to 100 µM. Our results demonstrate that NO-based screening using *S.* Enteritidis-infected HD11 cells is a viable tool to identify chemicals with anti-intracellular *Salmonella* activity. Using this method, we have shown H-89 has bacteriostatic activity against *Salmonella*, independent of host cell protein kinase A or Akt1 activity.

## Introduction

Salmonella enterica, comprising over 2500 serovars, are Gram-negative facultative intracellular bacteria responsible for diseases ranging from self-limiting gastroenteritis (non-typhoidal Salmonella) to life-threatening typhoid fever (serovar Typhi) in humans [1]. Non-typhoidal Salmonella, highly adaptive to divergent environments and commonly residing in the intestinal tracts of many animals, are one of the leading causes of foodborne illnesses worldwide [1]. Salmonella have evolved highly complex strategies to interact with host cells to circumvent immune defense mechanisms [2]. The type III secretion system (T3SS), which produces nearly 40 different virulence effectors, plays a major role in Salmonella invasion, survival, and replication inside the host cells [3]–[5]. Salmonella can gain entry into phagocytes such as macrophages via a combination of cell-initiated phagocytosis and bacteria-mediated invasion. Facilitated by these virulence effectors, internalized Salmonella survive and replicate within Salmonella-containing vacuoles (SCV) by manipulating the host cell to delay phagolysosomal maturation and therefore avoid exposure themself to lysosomal contents [3].

Recently, the increased antimicrobial resistance observed in Salmonella, i.e., multidrug-resistant (MDR) Salmonella, to growing numbers of commonly used antibiotics has become a major public health concern. Salmonella acquire multidrug resistance through mechanisms involving enzymatic inactivation of antimicrobial agents, reduction of cell surface permeability to antibiotics, activation of efflux pumps to remove drugs from the bacterial cells, and modification of the affinity of cellular targets of drugs through genetic mutations [6], [7]. Widespread use of antibiotics for growth promotion and disease control in food animal production may have contributed to the development of antibiotic resistant pathogens including many medically important Salmonella serotypes [6], [8]. Therefore, development of novel drugs with new modes of action is urgently needed. However, an effective screening method to identify novel pharmaceuticals that are capable of killing intracellular Salmonella remains to be developed.

Salmonella enterica serovar Enteritidis is one of the most prevalent Salmonella serovars in poultry and is frequently associated with clinical isolates in human salmonellosis [9], [10]. Studies show that S. Enteritidis is highly invasive and causes systemic infection and colonization of reproductive organs in poultry, which is a main source for contamination of eggs [11], [12]. Previous studies have shown that S. Enteritidis infection completely suppresses the production of nitric oxide (NO) in chicken macrophage HD11 cells, while heat-killed S. Enteritidis (HKSE) stimulates a large amount of NO production [13], [14]. Based on these observations, we conducted a study to test the hypothesis that the outcome of NO production in S. Enteritidis-infected HD11 cells depends on the viability of intracellular Salmonella and thus NO can serve as a biomarker to screen for pharmaceutical molecules with anti-intracellular Salmonella activity.

## Materials and Methods

### Reagents

Reagents for nitrite assays and antibiotics for bacterial culture were obtained from Sigma (St. Louis, MO, USA). Media for *Salmonella* culture were obtained from BD (Becton, Dickinson and Company, NJ, USA). Medium (Dulbecco’s Modified Eagles Medium, DMEM) for HD11 cell culture was obtained from Invitrogen (NY, USA) and medium additives were obtained from Sigma. Inhibitors used in this study were obtained from LC Laboratories (MA, USA), Cayman Chemical (MI, USA), and Santa Cruz Biotechnology (CA, USA).

### Bacterium

The primary poultry isolate of S. Enteritidis [15] used in this study was obtained from the National Veterinary Services Laboratory (Ames, IA, USA). A carbenicillin-novobiocin (C-N) resistant isolate was selected and stored in 75% trypticase soy broth (TSB)+25% sterile glycerol in aliquots of 1×10^9^ colony forming units (cfu) at –80°C until used. The S. Enteritidis used for infection of macrophage cells was cultured in TSB containing 100 µg/ml of C and 25 µg/ml of N overnight at 41°C and a 1∶10 dilution of the overnight culture, prepared in fresh TSB was incubated for 4 h to obtain bacteria that are in the exponential growth phase. The S. Enteritidis was collected, washed, and resuspended in PBS at ∼2×10^9^ cfu/ml. The viable cell concentration of S. Enteritidis was determined by colony counts on BD’s Difco’s xylose-lysine tergitol 4 (XLT4) agar plates containing C and N. HKSE was prepared by incubating the bacterial suspension in a 75°C water bath for 15 min and verified by overnight culture.

### Cell Line

The MC29 virus-transformed chicken macrophage cell line HD11 [16] was maintained in complete Dulbecco’s Modified Eagles Medium (DMEM) containing 10% chicken serum, antibiotics (100 U penicillin/ml and 100 µg streptomycin/ml), and 1.5 mM L-glutamine at 39°C, 5% CO_2_, and 95% humidity. Aliquots of cell suspension (2×10^6^ cells/ml) was seeded into each well at 500 µl/well in 24-well plates (BD) and allowed to grow to about 85% confluence (∼36 h) before used for infection.

### Intracellular Salmonella Viability Assay

Prior to infection, the culture medium was removed and the cells were washed once and replaced with 200 µl of plain DMEM (without chicken serum and antibiotics). Aliquots (50 µl) of Salmonella (at multiplicity of infection or MOI from 3 to 50) were added to each well in four replicates and incubated for 1 h at 39°C in a 5% CO_2_ humidified incubator. At 1 hour post infection (hpi), the infection medium was removed and the cells were washed once with plain DMEM, treated with 100 µg/ml of gentamicin sulfate in complete DMEM for 1 h to kill extracellular bacteria. After gentamicin treatment, infected cells were washed twice and cultured in complete DMEM containing 25 mg/ml of gentamicin sulfate for 24 h. At 24 hpi, infected cells were washed twice with PBS and lysed for 10 min in 1% Triton X-100 (in PBS). Serial 1∶10 dilutions of the lysates were plated onto XLT4 agar plates containing C and N and incubated at 41°C for 24 h. Colonies were counted to determine the cfu of intracellular viable bacteria.

### Nitric Oxide Production Assay

Nitrite, a stable metabolite of NO, produced by activated macrophages was measured by the Griess assay [17]. HD11 cells in 24-well plates were treated identically, in 4 replicates, with live *S*. Enteritidis (intracellular viability assay) or HKSE. After 24 hpi, aliquots of 100 µl culture supernatant from each well were transferred to the wells of a new flat-bottom 96-well plate and mixed with 50 µl of 1% sulfanilamide and 50 µl of 0.1% naphthylenediamine (both were prepared in 2.5% phosphoric acid solution) sequentially. After 10 min incubation at room temperature, the nitrite concentration was determined by measuring optical density (OD_595_) of each well using a SPECTRA MAX microplate reader (Molecular Devices, Sunnyvale, CA). Sodium nitrite (Sigma) was used as a standard to determine nitrite concentrations in the cell-free medium.

### Bacterial Growth Inhibition Assay

An overnight culture of S. Enteritidis was diluted 1∶20 in TSB containing various concentrations of inhibitor H-89 and incubated at 41°C in a 96-well optical plate. The optical densities at OD_600_ were measured at 2, 4, 8, and 24 h using a SPECTRA MAX microplate reader (Molecular Devices) to determine the growth of S. Enteritidis. At the end of 24 h incubation, viable S. Enteritidis in each treatment were determined by plating serial 1∶10 dilutions of the culture on the XLT4 agar plates containing C and N and incubated at 41°C for 24 h.

### Data Analysis

At least 3 independent experiments were conducted. Statistical differences were determined at the level of p<0.05 by One Way Analysis of Variance and the Tukey Test using SigmaStat software (Jandel Corp, San Rafael, CA, USA).

## Results

### Phagocytosis, Intracellular Survival, and Nitric Oxide Response in S. Enteritidis-infected HD11 Cells

**Figure 1:**
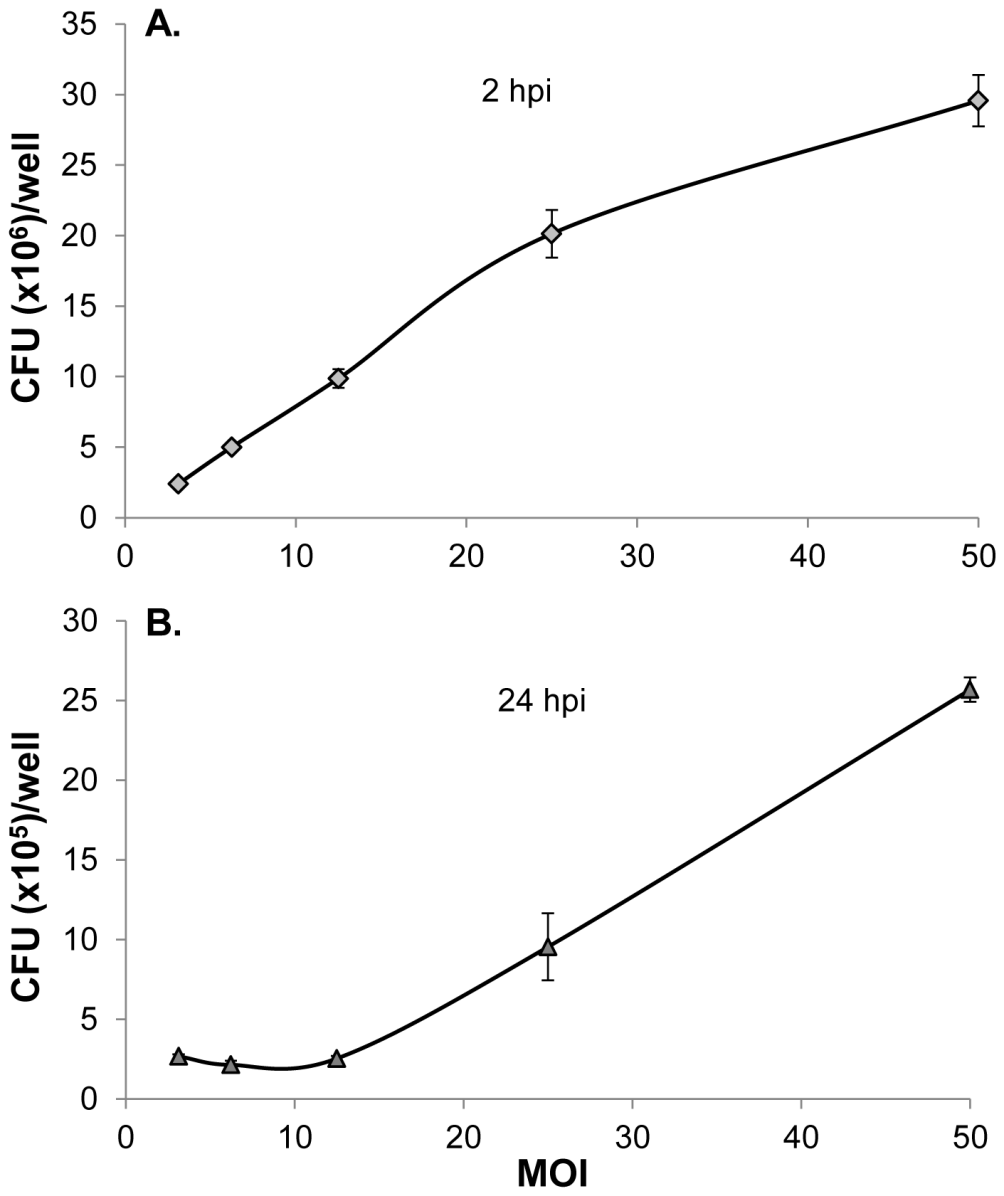
Intracellular *S*. Enteritidis in HD11 cells infected with various multiplicity of infection (MOI). HD11 cells were infected with *S*. Enteritidis at various MOI for 1 h in 24-well plates at 39°C in a 5% CO2 humidified incubator. At 1 hour post infection (hpi), extracellular *S*. Enteritidis were killed by incubation with media containing 100 µg/mL of gentamicin sulfate for 1 h. Intracellular *S*. Enteritidis (cfu) were determined at 2 and 24 hpi. A. Intracellular *S*. Enteritidis (cfu) at 2 hpi; B. Intracellular *S*. Enteritidis (cfu) at 24 hpi.

**Figure 2:**
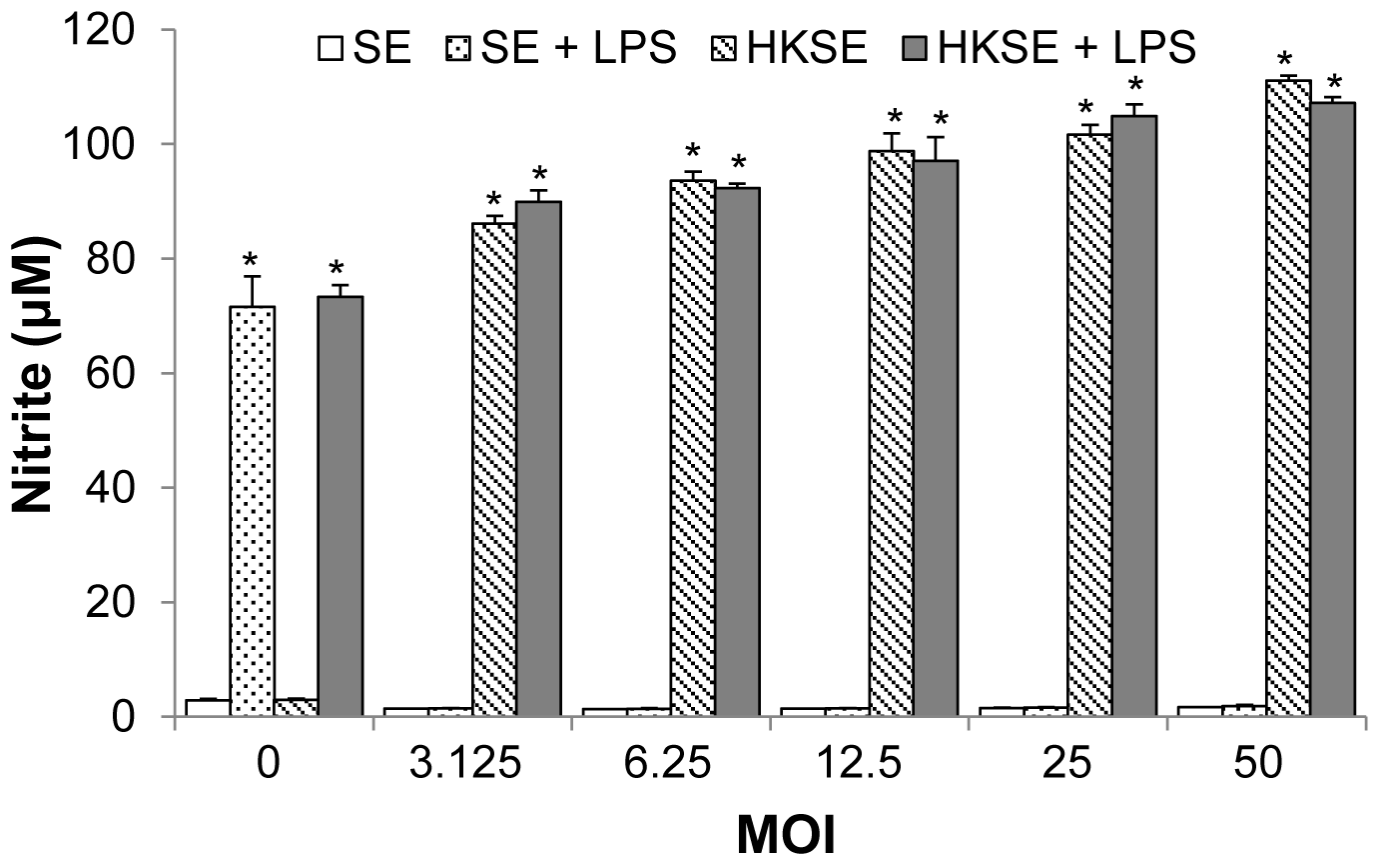
Effect of *S*. Enteritidis infection on nitric oxide (NO) production in HD11 cells. HD11 cells were infected with live *S.* Enteritidis (SE) for 1 h in 24-well plates at 39°C in a 5% CO2 humidified incubator. At 1 hour post infection (hpi), extracellular SE were killed by incubation with media containing 100 µg/mL of gentamicin sulfate for 1 h; the cells were washed and then cultured with or without lipopolysaccharide (LPS) at 0.2 µg/mL for an additional 22 h in a medium containing 20 µg/mL of gentamicin sulfate; and nitrite contents in cell culture media were determined. Treatment with heat-killed *S*. Enteritidis (HKSE) was performed identically as with live SE. The symbol (*) indicates that the difference between these groups and the control is statistically significant (p<0.05).

Phagocytosis and intracellular survival of S. Enteritidis in infected HD11 cells were examined at various MOI ranging from 3 to 50. HD11 cells phagocytized increased number of S. Enteritidis when the cells were infected with increased MOI from 3 to 50 (Figure 1A). Based on the number of HD11 cells, it was calculated that the MOI at 3 was required to ensure that each HD11 cell was infected by at least one S. Enteritidis bacterium. At 24 hpi, the numbers of viable intracellular S. Enteritidis were increased significantly when cells were infected with MOI at 25 and 50 (Figure 1B). To demonstrate that intracellular Salmonella suppress the NO response in infected HD11 cells, the cells were treated with both live S. Enteritidis and HKSE identically with MOI ranging from 3 to 50. After 24 h, production of NO in S. Enteritidis-infected HD11 cells was completely abolished regardless MOI; while HKSE-treated HD11 cells produced significant amounts of NO in all treatments at various MOI (Figure 2). To further verify that Salmonella infection suppresses NO production in HD11 cells, both infected (at various MOI) and non-infected HD11 cells were stimulated with Salmonella lipopolysaccharide (LPS, Invitrogen). Again, LPS (0.2 µg/ml) stimulated a significant production of NO in uninfected HD11 cells, while LPS-induced NO productions in Salmonella-infected HD11 cells were completely suppressed (Figure 2). In contrast to live S. Enteritidis, HKSE treatments had no effect on LPS-induced NO production (Figure 2). These results clearly showed that intracellular S. Enteritidis suppress NO response in HD11 cells. For in vitro bacterial infection experiment, high MOI ranging from 10 to 100 are commonly used to ensure the homogeneous infection in the cell population. In the present study, a high MOI was desired to achieve a maximal load of intracellular bacteria in HD11 cell for assessing the killing by the inhibitors. Therefore, the MOI at 50 was used in all our experiments.

### Effect of Pharmaceutical Inhibitors on NO Production of HD11 Cells Infected with S. Enteritidis

The observation that intracellular S. Enteritidis suppressed the NO response in HD11 cells provided the basis of our hypothesis that treatment of infected HD11 cells with chemicals that kill the intracellular Salmonella should relieve the suppression and restore the NO production. Knowing that host cell kinases play a critical role in the survival of intracellular Salmonella [18], a group of pharmaceutical inhibitors of various kinases were selected for testing their effects on NO production in S. Enteritidis-infected HD11 cells. Among these inhibitors, a protein kinase A inhibitor, H-89, was found to reverse the suppression of Salmonella on NO production in infected HD11 cells; while treatments with other inhibitors showed no such effect (Table 1).

**Table 1:** Effect of kinase inhibitors on nitric oxide production in Salmonella-infected chicken macrophage HD-11 cells.

Inhibitors	Concentration (µM)	Nitrite (µM)
SE-infected HD11 control0.9±0.4	–	0.9±0.4
Rapamycin (mTOR inhibitor)	10	0.8±0.1
	50	0.2±0.1
Rp-cAMPS (cAMP-dependent protein kinase (PKA) inhibitor)	20	0.9±0.2
	100	0.5±0.1
HA-1077 (Rho kinase inhibitor)	10	0.8±0.2
	50	0.8±0.1
KN-93 (Ca2+/calmodulin-dependent kinase II (CaMK II) inhibitor)	20	0.6±0.1
	100	2.7±0.8
Akti-1/2 (Akt1/2 (PKB) inhibitor)	10	2.3±1.0
	50	1.7±0.6
H-89 (cAMP-dependent protein kinase (PKA) inhibitor)	10	2.8±0.6
	30	98.0±2.4*
DRB (Casein Kinase II (CK2) Inhibitor)	20	0.5±0.2
	100	0.5±0.1
PD98059 (MAPK/ERK1/2 kinase inhibitor)	10	0.4±0.1
	50	0.3±0.2
SB203580 (p38-MAPK inhibitor)	10	0.4±0.2
	50	0.3±0.2
SP600125 (c-Jun N-terminal kinase (JNK) inhibitor)	10	0.7±0.1
	50	0.3±0.1
Tamoxifen Citrate (protein kinase C (PKC) inhibitor)	20	0.4±0.1
	100	1.0±0.1

The HD-11 cells were infected with S. Enteritidis as described in the Materials and Methods and followed by treatment with various inhibitors at concentrations indicated. Nitrite in the culture media was measured at 24 hpi. Data are means ± standard deviations. The symbol (*) indicates the differences between the control and the treatments are statistically significant (P≤0.05).

### Relationship between NO Production and Intracellular Viability of S. Enteritidis in H-89 Treated HD11 Cells

**Figure 3:**
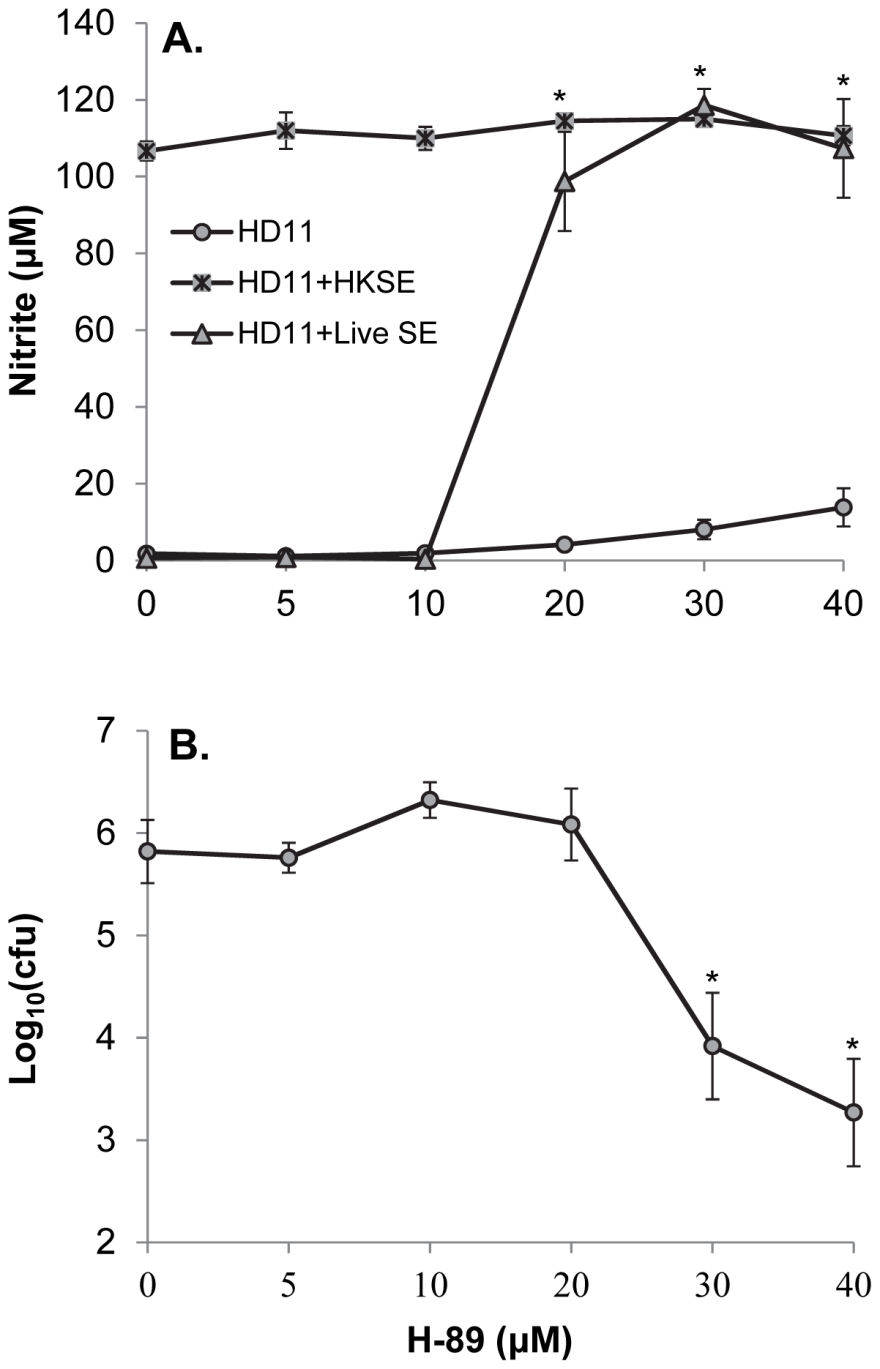
Effect of H-89 treatment on nitric oxide (NO) production in *S*. Enteritidis-infected HD11 cells and the intracellular survival of *S*. Enteritidis. A. Effect of H-89 treatment on NO production in *S*. Enteritidis-infected HD11 cells. HD11 cells were infected with live *S*. Enteritidis (SE) for 1 h in 24-well plates at 39°C in a 5% CO2 humidified incubator. At 1 hour post infection (hpi), extracellular SE were killed by incubation with media containing 100 µg/mL of gentamicin sulfate for 1 h; the cells were washed and then cultured in media containing various concentrations of H-89 and 20 µg/mL of gentamicin sulfate for an additional 22 h; and nitrite contents in cell culture media were determined. Treatment with heat-killed *S*. Enteritidis (HKSE) was performed identically as with live SE. B. HD11 cells were infected with live SE as described above and at 24 hpi, intracellular viable SE [Log10 (cfu)] were counted. The symbol (*) indicates that the difference between these groups and the respective controls is statistically significant (p<0.05).

The above screening results indicated H-89 at 30 µM dramatically reversed the suppressive effect of S. Enteritidis on the production of NO in infected HD11 cells. To verify that this reversal of NO production by H-89 was a result of increased killing of intracellular S. Enteritidis, the viable numbers of intracellular Salmonella at 24 hpi were determined in infected HD11 cells treated with various concentrations of H-89. The NO productions were also measured simultaneously. The results showed that increased NO production was correlated with decreased intracellular Salmonella viability. The NO production in S. Enteritidis-infected HD11 cells was increased significantly (p≤0.05) with H-89 treatment at or above 20 µM (Figure 3A), while the number of viable intracellular Salmonella was reduced significantly (p≤0.05) when the cells were treated with H-89 at or above 30 µM (Figure 3B). These results suggest that H-89 metabolically inhibits intracellular S. Enteritidis and that this inhibition facilitates the killing of intracellular Salmonella.

### Inhibition of S. Enteritidis Growth in Medium by H-89

**Figure 4:**
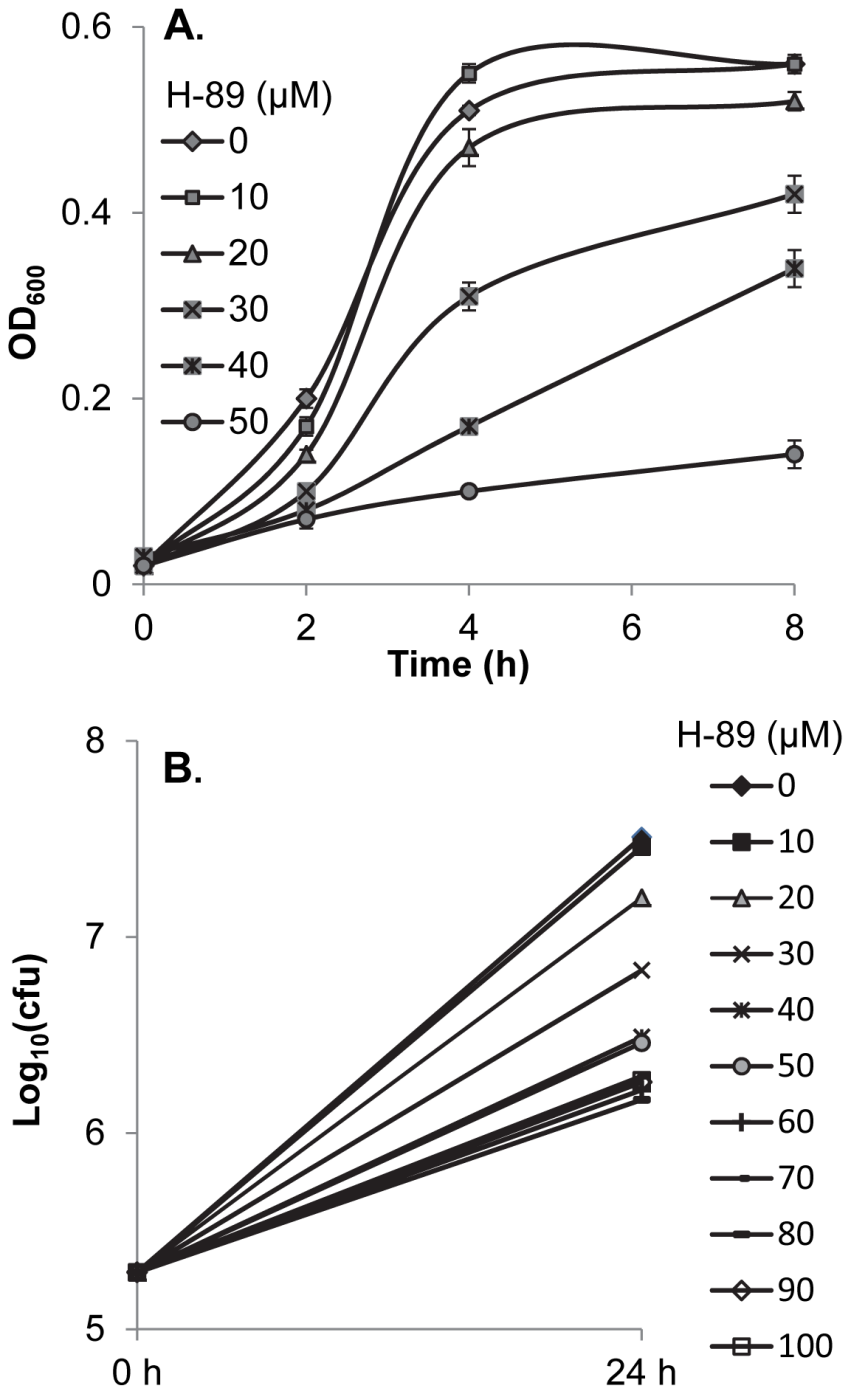
Effect of H-89 on *S*. Enteritidis growth in trypticase soy broth (TSB). A. The growth curves of *S*. Enteritidis in TSB containing various concentrations of H-89. An overnight culture of *S*. Enteritidis was diluted 1∶20 in TSB medium containing various concentrations of H-89 and incubated at 41°C in a 96-well optical plate. The optical densities at OD600 were measured at 2, 4, 8, and 24 h using a microplate reader to determine the growth of *S*. Enteritidis. B. The viable *S*. Enteritidis in culture containing various concentrations of H-89. At the beginning (2 hpi) and the end of incubation (24 hpi), viable *S*. Enteritidis [Log10 (cfu)] in each treatment were determined by plating serial 1∶10 dilutions of the culture on the Difco’s xylose-lysine tergitol 4 (XLT4) agar plates and incubated at 41°C for 24 h.

H-89 is a cell permeable compound and our results indicated that it has an antagonistic effect on intracellular S. Enteritidis. However, it was not clear whether H-89 asserts its anti-intracellular S. Enteritidis activity via an effect on the host cell or directly on intracellular S. Enteritidis. Therefore, the effect of H-89 on the growth of S. Enteritidis in TSB was evaluated. The growth curves (Figure 4A) clearly demonstrated a dose-dependent antagonistic effect of H-89 on S. Enteritidis. The maximum inhibition of the bacterial growth by H-89 was achieved at 50 µM. However, S. Enteritidis was not killed by H-89 treatments, since there was appreciable growth in all treatment groups as shown in the viability assay after 24 h culture (Figure 4B). These results indicated that H-89 effectively retarded the growth of S. Enteritidis rather than killed the bacterium, suggesting that H-89 has anti-Salmonella bacteriostatic properties that diminish the ability of S. Enteritidis to resist the bactericidal activities of HD11 cells.

## Discussion

Macrophages are important innate immune cells that play a central role in the first line of defense against microbial infection in which they detect, phagocytize, and produce microbicidal substances, including reactive radical oxygen species (ROS), NO, lysozyme, and proteolytic enzymes, to kill the infectious agents. NO is a multi-functional mediator with diverse physiological and pathological roles in vasodilatation, neurotransmission, and host defense against infectious agents and tumors [19]. In macrophages, NO is synthesized from L-arginine, oxygen and NADPH by inducible nitric oxide synthase (iNOS) in response to stimulation by microbial products [20]. The NO response to microbial stimulation is an important innate immune function of macrophages and plays a critical role in controlling the proliferation of intracellular bacterial pathogens such as Salmonella Typhimurium [21]–[23]. To counter the adversary effect of NO, Salmonella T3SS secretes effector proteins to suppress iNOS activity [24] and prevent iNOS-containing vesicle trafficking to phagosomes, hence limiting exposure of Salmonella to reactive nitrogen species (RNS) [25]. Additionally, Salmonella produce enzymes, including flavohemoglobin Hmp, flavorubredoxin NorV, and cytochrome c nitrite reductase NrfA, which can detoxify NO under varying environmental conditions [26], [27]. Therefore, the outcome of NO production in infected macrophages will largely depend on the metabolic state of the intracellular Salmonella and the role of NO in Salmonella pathogenesis remains controversial [28].

In the present study, the NO production in HD11 cells was highly responsive to HKSE stimulation, but was completely lacking in the cells infected with live S. Enteritidis. The results clearly indicate that intracellular S. Enteritidis suppressed the NO production in the infected HD11 cells and this suppression was relieved when the bacteria were metabolically inactive (e.g. HKSE). The S. Enteritidis infection has also abolished LPS-stimulated NO production in HD11 cells, further demonstrating that intracellular S. Enteritidis can effectively suppress the host cell’s NO response. A recent study shows that not all Salmonella strains are equally capable of suppressing the NO response in the infected HD11 cells [14]. Among the tested poultry serotype isolates, S. Enteritidis completely abolishes the NO response, followed by S. Typhimurium to a lesser extent; while serotypes S. Heidelberg, S. Kentucky, and S. Senftenberg lack the ability to suppress the NO response in respectively infected HD11 cells. The ability to totally suppress the host cell’s NO response allows S. Enteritidis to avoid the detrimental effect of oxidative and nitrosative stress of NO and its derivatives. These results, however, seem to disagree with many previously studies which show that infections with S. Typhimurium and S. Enteritidis stimulate NO production in chicken macrophages [29]–[31]. We are not sure the cause of this discrepancy. However, we believe that different strains of bacteria and the preparation of bacteria may have contributed to the discrepancy. In our studies, consistent results were achieved by using bacteria that were freshly harvested after 4 h culture. Therefore, the growth phase of the bacteria may also be a factor affecting the outcome of the results.

The virulence factors of Salmonella are known to facilitate the interaction of the bacteria with host cells through remodeling of the host cell cytoskeleton, altering cellular trafficking, modulating kinase activities and immune responses to allow them to gain entry into host cells, and to survive and replicate within host cells [18], [32]. Increasing evidence indicates that the host cellular kinase network is an important target of Salmonella virulence factors and many of these kinases play critical roles in Salmonella pathogenesis. A recent study using a phosphoproteomic approach revealed that the host kinase network is extensively manipulated by Salmonella infection and that the Salmonella effector protein SopB, a phosphoinositide phosphatase, plays a major role in manipulation of the host cell phosphorylation [33]. Specifically, Salmonella SopB and SopE/E2 (guanine nucleotide exchange factors) activate the Rho family guanosine triphosphatases (GTPases) and the cell division control protein 42 (Cdc42), which play a central role in the actin cytoskeleton remodeling and invasion [34]. SopB also activates phosphatidyl inositide 3 kinase (PI3 K)/Akt kinase signaling pathway, which is critical for intracellular survival [35], [36]. A recent study indicates that activation of the host cell PI3 K, Akt, and Rac-1 is required for Salmonella outer membrane protein Rck-mediated invasion [37]. Salmonella AvrA, an acetyltransferease, acts to suppress c-Jun N-terminal kinase (JNK) activation, preventing macrophage death and facilitating bacterial dissemination [38]. Additionally, AvrA inhibits mitogen-activated protein kinases (MAPK) and suppresses NF-κB activation, thereby suppressing the inflammatory responses in Drosophila and yeast models [39], [40]. Salmonella SpvC as a phosphothreonine lyase dephosphorylates and inactivates extracellular signal-regulated protein kinases 1 and 2 (ERK1/2), possibly also p38 MAPK and JNK, and suppresses the host immune response [41], [42]. These results underscore the importance of host cell kinases in Salmonella pathogenesis. Therefore, in the present study various kinase inhibitors were tested for their effect on the survival of intracellular S. Enteritidis.

Among kinase inhibitors tested, H-89 was the only one that showed antagonistic activity against intracellular S. Enteritidis. H-89 enhanced the killing of intracellular S. Enteritidis and reversed the suppression of the NO response in S. Enteritidis-infected HD11 cells. The effective concentration of H-89 required for reversing the suppression of NO production in infected HD11 cells was 20 µM which was lower than the concentration (30 µM) needed to induce significant reduction of intracellular S. Enteritidis. This observation suggests that H-89 at 20 µM is sufficient to inhibit the metabolic function of S. Enteritidis and reverse the S. Enteritidis-mediated suppression of NO production in infected HD11 cells. The in vitro culture also indicated that H-89 at 20 µM starts to cause significant growth retardation of S. Enteritidis, suggesting that H-89 acts identically both intracellularly and extracellularly. More importantly, our results reveal that the nature of antagonistic activity of H-89 against S. Enteritidis is bacteriostatic rather than bactericidal, as H-89 only arrests the growth of S. Enteritidis. These observations suggest that H-89 most likely deteriorates the fitness of S. Enteritidis, reducing the ability to resist the bactericidal activities of HD11 cells, and eventually leading to an increased killing of intracellular S. Enteritidis. H-89 was originally identified as a selective inhibitor of cAMP dependent protein kinase (PKA). Recently, it was also found to broadly inhibit many other kinases [43]. Previously, H-89 was reported to reduce S. typhimurium survival in the murine macrophage cell line Raw264.7 and the reduction was thought to be caused by inhibition of the host protein kinase A [44]. A recent study shows that, at 10 µM, H-89 is able to inhibit intracellular S. typhimurium growth in the human breast cancer cell line MCF7 and the inhibition of host cell’s Akt1 by H-89 is most likely the attributing factor [35]. Although, H-89 at 10 µM was reported to have no effect on S. typhimurium growth in LB medium, higher concentrations were not tested [35]. Our study supports the antibacterial activity of H-89 reported on S. typhimurium in infected MCF7 cells [35]; however, a different mechanism may be involved in H-89 induced killing of intracellular S. Enteritidis in HD11 cells. The involvement of host cell’s Akt1 inhibition was not confirmed in H-89 induced killing in S. Enteritidis infected HD11 cells, since treatment with the selective Akt1/2 kinase inhibitor (Akti-1/2) failed to reproduce the same antagonistic effect observed in H-89. On the contrary, our results attribute the increased killing of intracellular S. Enteritidis to the direct bacteriostatic effect of H-89 on the bacterium, which was clearly demonstrated in the in vitro culture experiments. The mechanism of the bacteriostatic activity of H-89 against S. Enteritidis is not clear; however, it is possible that H-89 may have also inhibited S. Enteritidis kinases.

Our study demonstrated that using NO production in S. Enteritidis infected HD11 cells as a biomarker is a viable tool to screen chemicals with antibacterial activity against intracellular Salmonella. This platform is inexpensive and can be easily adapted to a 96-well assay format. Measuring NO production is a simple procedure and is much less time consuming and labor intensive than the bacterial culture cfu assay to determine the surviving intracellular S. Enteritidis. Additionally, the strain of S. Enteritidis used in our method is a wild type field isolate, which should be preferable than using genetically modified strains. The advantage of this method is in its ability to identify chemicals with anti-intracellular Salmonella properties. The effectiveness of this method is demonstrated by the discovery, for the first time, of H-89 having bacteriostatic activity against Salmonella. Although S. Enteritidis and the chicken macrophage cell line HD11 are used in this assay format, the potential application of newly identified anti-Salmonella molecules is certainly not limited to chickens.
